# Infra‐tubercle osteotomy preserves coronal alignment and reduces anterior laxity compared to retro‐tubercle technique in revision anterior cruciate ligament reconstruction with slope correction

**DOI:** 10.1002/ksa.70250

**Published:** 2025-12-26

**Authors:** Romir Patel, Ahmed Mabrouk, Souvik Paul, Christophe Jacquet, Matthieu Ollivier

**Affiliations:** ^1^ Department of Orthopedic Surgery, APHM, CNRS, ISM, Institute of Movement Sciences, Sainte‐Marguerite Hospital Aix Marseille University Marseille France; ^2^ Department of Orthopaedic Surgery Massachusetts General Hospital Boston Massachusetts USA; ^3^ Trauma and Orthopaedics Department Basingstoke and North Hampshire Hospitals Basingstoke UK

**Keywords:** ACL revision, coronal alignment, high tibial osteotomy, posterior tibial slope, survivorship

## Abstract

**Purpose:**

To compare infra‐tubercle (IKO) versus retro‐tubercle (RKO) slope‐reducing osteotomy performed with revision anterior cruciate ligament reconstruction (ACLR) on survivorship, anterior laxity, alignment, union, complications and patient‐reported outcome measures (PROMs).

**Methods:**

Retrospective comparative cohort at a tertiary centre including 107 consecutive revision ACLR + slope‐reducing osteotomy cases (IKO *n* = 50; RKO *n* = 57). Primary outcomes were Kaplan–Meier survivorship for (1) revision‐only and (2) global failure (earliest of revision or instrumented anterior laxity > 5 mm). Secondary outcomes included Rolimeter side‐to‐side laxity at 2 months/1 year/2 years, radiographic posterior tibial slope (ΔPTS) and hip–knee–ankle angle (ΔHKA), union time, complications/reoperations and knee injury and osteoarthritis outcome score (KOOS). Between‐group comparisons used *t*‐tests/*χ*²; survival was compared by the log‐rank test. Significance *p* < 0.05.

**Results:**

At mean follow‐up of 27.9 ± 4.4 months, crude revision rates were 5.26% (3/57; 95% confidence [CI]: 1.8%–14.4%) for IKO versus 20.0% (10/50; 95% CI: 11.2%–33.0%) for RKO (*p* = 0.048). Five‐year Kaplan–Meier survivorship estimates showed no statistically significant differences at all endpoints. IKO demonstrated significantly lower instrumented laxity at all time points: 2.4 ± 2.1 versus 3.3 ± 2.0 mm at 2 months (*p *= 0.034), 3.5 ± 2.3 versus 4.8 ± 2.7 mm at 1 year (*p *= 0.010), and 4.0 ± 2.9 versus 5.8 ± 4.2 mm at 2 years (*p* = 0.013). Coronal alignment changes differed significantly between groups (ΔHKA + 0.45° ± 0.58° for IKO vs. −1.32° ± 1.20° for RKO, *p* < 0.001). PTS reduction was comparable between groups (ΔPTS −8.20° ± 1.61°, 95% CI: −8.6 to −7.8 vs. −7.90° ± 1.74°, 95% CI: −8.4 to −7.4, *p* = 0.35). Hardware removal was more frequent following IKO (38.8% vs. 17.2%, *p* = 0.016). Union was achieved universally in both groups, though healing time was longer after IKO (4.0 ± 0.9 vs. 3.2 ± 0.6 months, *p* < 0.0001).

**Conclusion:**

In revision ACLR for elevated PTS, IKO and RKO yield comparable survivorship and PROM gains. IKO demonstrated better preservation of coronal‐plane alignment and lower residual anterior laxity, at the trade‐off of more frequent elective hardware removal and slightly longer time to union. These data can inform technique selection and counselling.

**Level of Evidence:**

Level III, retrospective comparative cohort.

**Abbreviations**
ACL: anterior cruciate ligament
ACLR: anterior cruciate ligament reconstruction
ACL‐RSI: anterior cruciate ligament–return to sport after injury (scale)
ACW‐HTO: anterior closing‐wedge high tibial osteotomy
ADL: activities of daily living
AP: anteroposterior (radiograph)
BMI: body mass index
BTB: bone–patellar tendon–bone (graft)
HKA: hip–knee–ankle angle
HS: hamstring tendon (graft)
HTO: high tibial osteotomy
ICC: intraclass correlation coefficient
IKDC: International Knee Documentation Committee (subjective knee form)
IKO: infra‐tubercle osteotomy
KOOS: knee injury and osteoarthritis outcome score
KT‐1000: KT‐1000 arthrometer
K‐wire: Kirschner wire
LET: lateral extra‐articular tenodesis
MCL: medial collateral ligament
MRI: magnetic resonance imaging
PCL: posterior cruciate ligament
PLC: posterolateral corner
PROMs: patient‐reported outcome measures
PTS: posterior tibial slope
QT: quadriceps tendon (graft)
RKO: retro‐tubercle osteotomy
ROM: range of motion
SD: standard deviation
SR‐HTO: slope‐reducing high tibial osteotomy

## INTRODUCTION

Revision anterior cruciate ligament reconstruction (ACLR) remains challenging with failure and residual laxity rates exceed those after primary surgery. Among anatomic risk factors, elevated posterior tibial slope (PTS) augments anterior tibial translation through increased shear loading on the graft and is consistently associated with both primary ACL injury and graft failure after ACLR [3, 4, 8]. Recent meta‐analyses confirm that patients with higher PTS have a significantly greater risk of ACL injury and ACLR graft failure, reinforcing PTS as a clinically meaningful modifier of stability [4].

Accordingly, sagittal‐plane correction with an anterior closing‐wedge high tibial osteotomy (ACW‐HTO) has emerged as a key adjunct in high‐slope, instability‐prone knees, particularly in the revision setting. Two sagittal osteotomy strategies are commonly employed: retro‐tubercle and infra‐tubercle approaches. The infra‐tubercle approach places the osteotomy wedge distal to the tubercle, potentially isolating correction to the sagittal plane while preserving the patellar tendon insertion [10, 14]. In contrast, the retro‐tubercle approach may inadvertently influence frontal‐plane alignment due to its ascending posterior cuts [2]. Emerging comparative data suggest that infra‐tubercle ACW‐HTO may provide more precise sagittal correction while better preserving frontal‐plane alignment and patellar height, yet comparative clinical evidence in the revision ACLR setting remains limited [14].

This study compared infra‐tubercle and retro‐tubercle ACW‐HTO performed with revision ACLR, evaluating survivorship, instrumented anterior laxity, radiographic alignment, union, complications and patient‐reported outcomes. It was hypothesised that survivorship would be similar between techniques, with the infra‐tubercle approach yielding decreased residual anterior laxity.

## METHODS

### Study design and setting

This study received Institutional Review Board approval (IRB #Cse_pads25_043_dgr), and the requirement for informed consent was waived given the retrospective design using de‐identified data. A retrospective comparative cohort study at a single tertiary centre included 107 consecutive adults (2016–2024) who underwent revision ACLR with concurrent slope‐reducing ACW‐HTO, performed by one senior surgeon.

**Figure 1:**
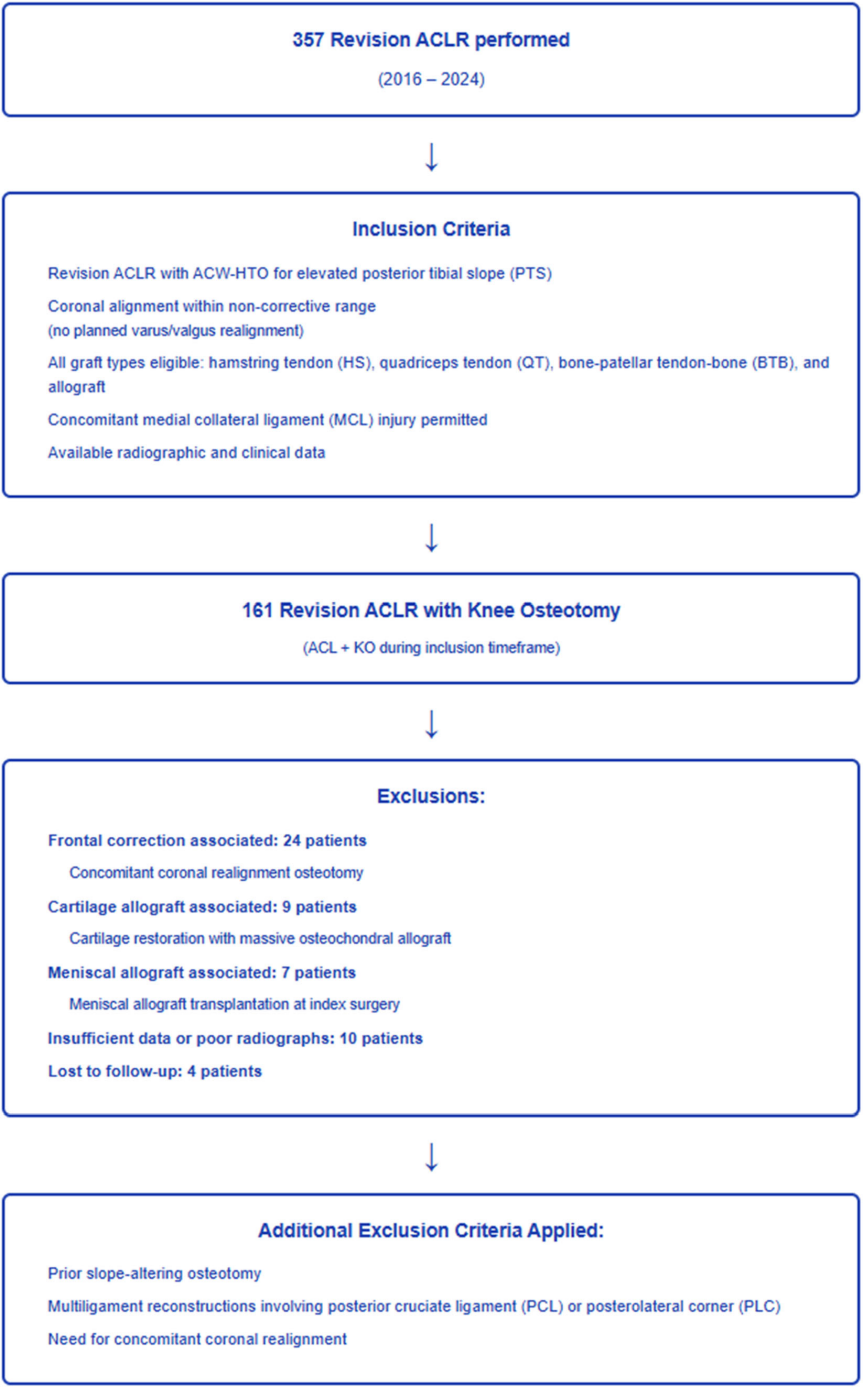
Flow‐chart describing patient selection. ACL, anterior cruciate ligament; ACLR, anterior cruciate ligament reconstruction; ACW‐HTO, anterior closing‐wedge high tibial osteotomy; KO, knee osteotomy.

Inclusion criteria were revision ACLR with ACW‐HTO for elevated PTS and coronal alignment within a non‐corrective range (no planned varus/valgus realignment). All graft types were eligible: hamstring tendon (HS), quadriceps tendon, and bone–patellar tendon–bone (BTB). Concomitant medial collateral ligament (MCL) injury was permitted. Exclusion criteria were prior slope‐altering osteotomy, need for concomitant coronal realignment osteotomy, meniscal allograft transplantation at index surgery, cartilage restoration with massive osteochondral allograft, and multiligament reconstructions involving the posterior cruciate ligament (PCL) or posterolateral corner (PLC) (Figure 1).

Baseline demographic and clinical characteristics are presented in Table 1. Groups were comparable for age, BMI, sex, follow‐up duration, preoperative PTS, graft type and prevalence of meniscal and chondral lesions. Prior meniscectomy was significantly more common in the infra‐tubercle osteotomy (IKO) group (51.0% vs. 27.6%, *p* = 0.01).

**Table 1:** Baseline demographics and clinical characteristics.

Variable	IKO (*n* = 50)	RKO (*n* = 57)	*p* value
Demographics			
Age (years)	26.4 ± 6.3	26.1 ± 4.7	0.770
BMI (kg/m²)	23.6 ± 3.8	24.6 ± 4.0	0.210
Female sex (%)	53.1	50.0	0.750
Follow‐up (months)	27.4 ± 4.3	28.5 ± 4.5	0.210
Surgical history			
Previous LET (%)	8.2	12.1	0.510
Number of prior ACL revisions (%)			0.340
‐ 1 revision	2.0	0.0	
‐ 2 revisions	73.5	82.8	
‐ ≥3 revisions	24.5	17.2	
Previous meniscectomy (any) (%)	51.0	27.6	0.010*
Baseline imaging			
Preoperative PTS (°)	13.7 ± 1.1	13.3 ± 1.3	0.060
Index surgery details			
BTB graft (%)	61.2	60.3	0.930
Meniscal pathology			
Medial meniscus lesion (%)	89.8	91.4	1.000
‐ Ramp lesion	28.6	29.3	0.930
‐ Meniscectomy performed	44.9	25.9	0.040*
Lateral meniscus lesion (%)	75.5	72.4	0.720
‐ Root/posterior radial	30.6	41.4	0.250
‐ Meniscectomy performed	22.4	12.1	0.150
Chondral status			
Any full‐thickness lesion (grade 3–4) (%)	18.4	25.9	0.350

Abbreviations: ACL, anterior cruciate ligament; BMI, body mass index; BTB, bone–patellar tendon–bone (graft); IKO, infra‐tubercle osteotomy; LET, lateral extra‐articular tenodesis; PTS, posterior tibial slope; RKO, retro‐tubercle osteotomy.

* Indicates statistical significance (*p* < 0.05).

### Surgical techniques

The choice between infra‐tubercle and retro‐tubercle osteotomy (RKO) was made by the senior surgeon based on anatomic considerations and surgical preference, which evolved over the study period. Early in the study period (2016–2019), the retro‐tubercle technique was preferentially used based on familiarity with published techniques. As experience with the infra‐tubercle technique increased and preliminary data suggested potential advantages in coronal alignment preservation, the infra‐tubercle technique was increasingly adopted (2020–2024).

A one‐ versus two‐stage approach was chosen based on tunnel enlargement/malposition and bone stock. Enlarged tunnels (>15 mm) were curetted and bone‐grafted; revision ACLR was delayed until radiographic evidence of graft incorporation/osteotomy union when staged. For survival analysis, time zero was defined as the index osteotomy procedure for all cases, regardless of staging. New anatomic femoral and tibial tunnels were created at the time of revision. Graft choice and fixation followed standard practice based on activity level, prior graft and tunnel size. Lateral extra‐articular tenodesis (LET) was performed if not previously done.

**Figure 2:**
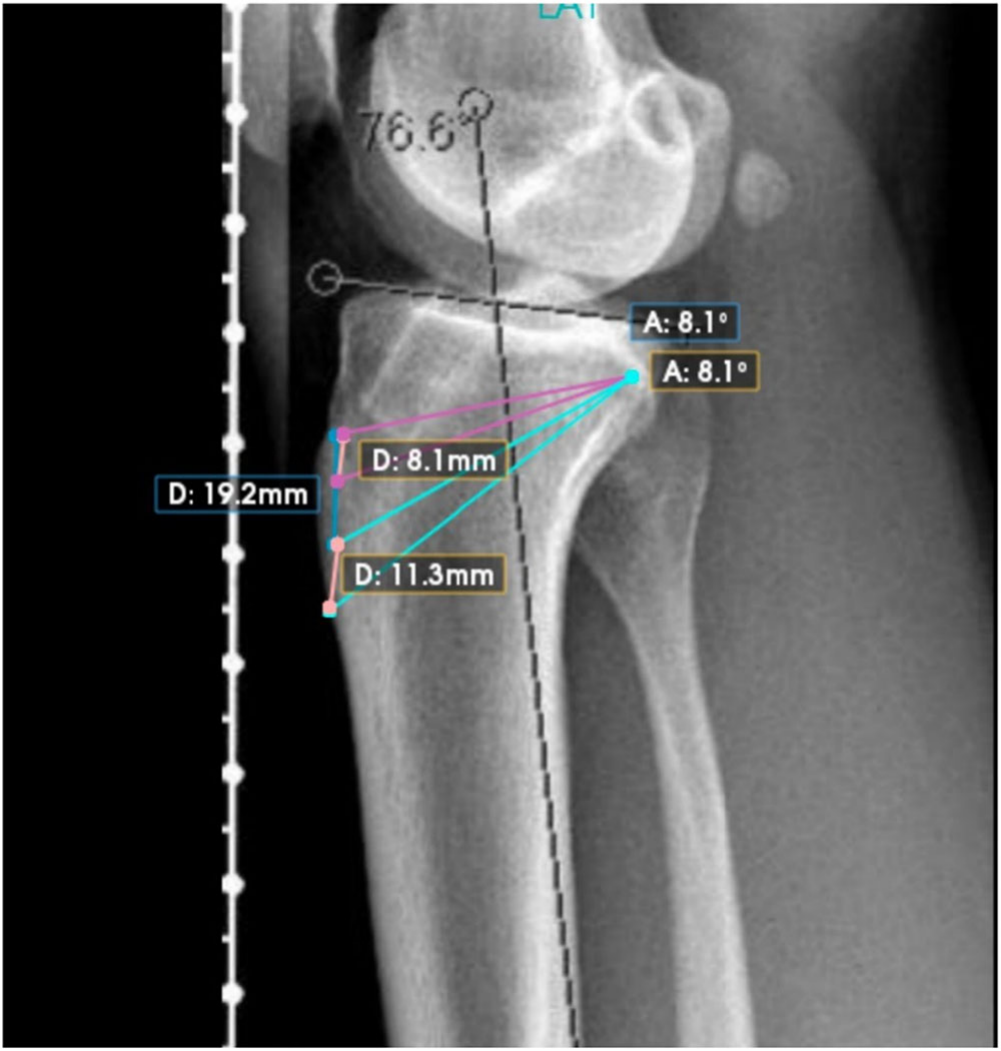
Lateral knee radiograph illustrating anterior closing‐wedge slope–reducing osteotomies at two levels relative to the tibial tubercle. Pink lines = retro‐tubercle wedge cuts (wedge level proximal to the tibial tubercle). Turquoise lines = infra‐tubercle wedge cuts (wedge level distal to the tibial tubercle).

The IKO approach was performed through a medial incision distal to the tibial tubercle; anterior and posterior cuts were made distal to the tubercle under Kirschner wire (K‐wire) guidance. After wedge removal, the osteotomy was compressed in full extension and fixed with a six‐hole locking plate (Figure 2) [12]. The RKO approach was performed via a midline incision; cuts were made posterior to the tibial tubercle with ascending posterior cuts toward the posterior cortex. Protective K‐wires were used to avoid hinge fracture. After wedge removal, axial compression in extension and six‐hole plate fixation were performed (Figure 2) [8].

Postoperative rehabilitation was identical for both groups. Immediate radiographs confirmed alignment and hardware. A PCL‐style brace was locked in −10° extension for approximately 3 weeks with nonweight‐bearing and crutch assistance. Partial weightbearing began at approximately 4 weeks and progressed to full weightbearing by approximately 6 weeks as tolerated. Range of motion was initiated in weeks 1–3 (goal 0°–90°). For staged cases, revision ACLR was performed after radiographic union of the osteotomy and adequate tunnel healing.

### Imaging protocols

**Figure 3:**
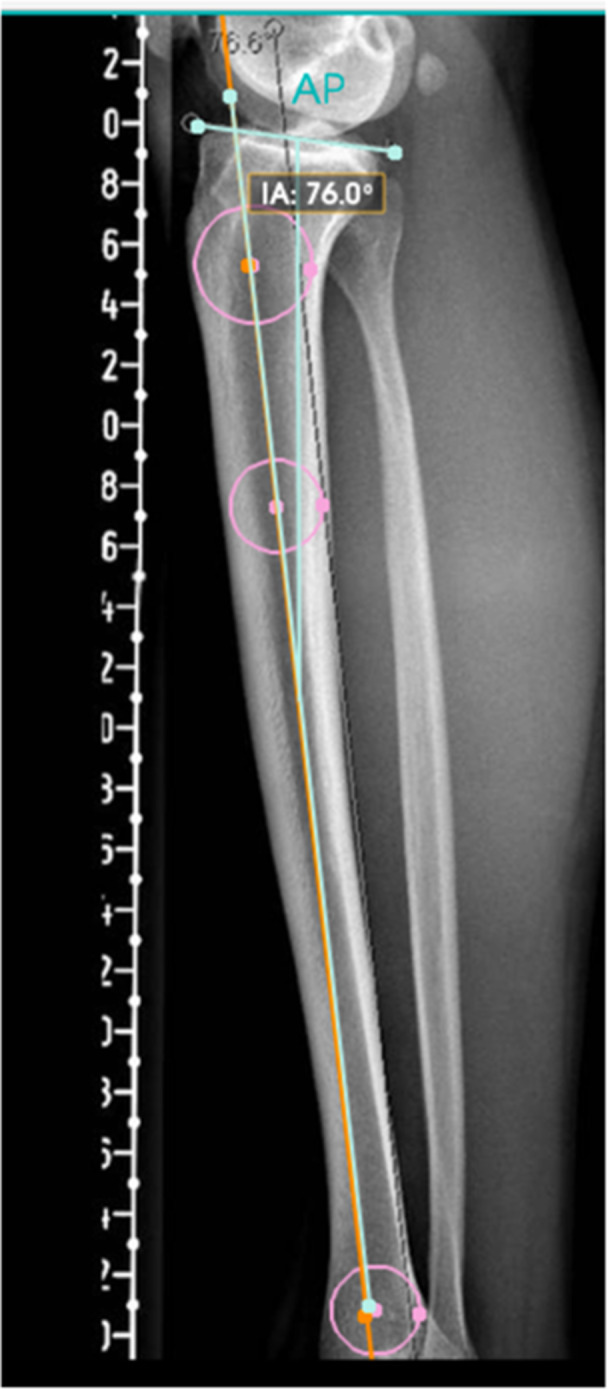
Three‐circle method to determine posterior tibial slope (PTS). Three best‐fit circles are placed along the proximal tibial diaphysis, and a line connecting their centres defines the longitudinal tibial axis. PTS is measured as the angle between the tibial plateau tangent and a line perpendicular to this axis. Interligne angle (IA) shown by the software is the angle between the plateau tangent and the tibial axis; thus PTS = 90°−IA.

On standardised true lateral knee radiographs (posterior femoral condyles superimposed), the tibial anatomic axis was defined with the circle method: three best‐fit circles were placed along the proximal tibial diaphysis, and a line connecting their centres defined the longitudinal axis. PTS was the angle between the tibial plateau tangent and a line perpendicular to this axis (Figure 3) [11].

**Figure 4:**
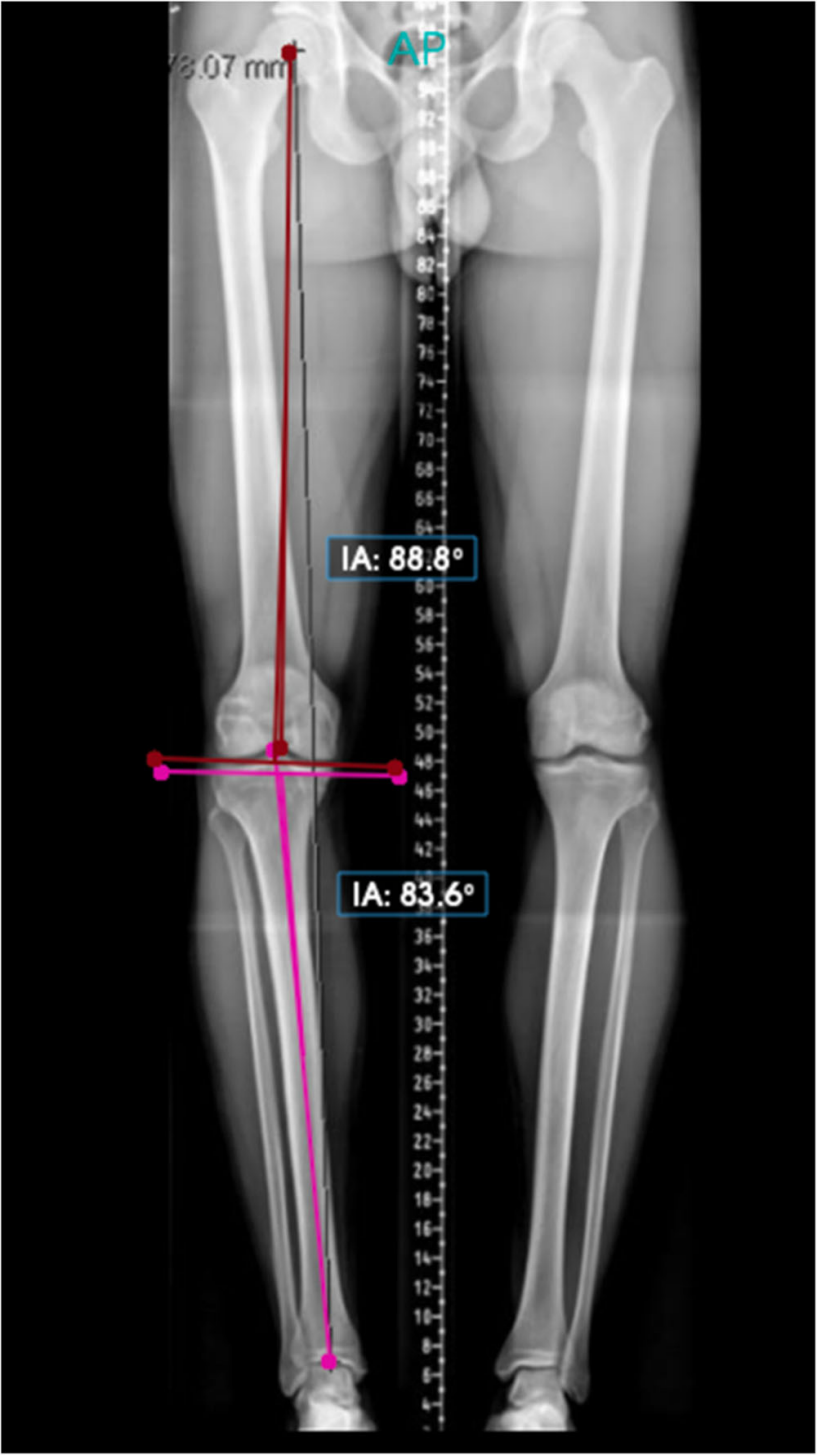
Long‐leg standing radiographs demonstrating hip–knee–ankle angle (HKA) measurement. HKA is measured as the mechanical femorotibial angle: the angle between the mechanical femoral axis (line from femoral head centre to knee centre, defined as midpoint between tibial spines) and the mechanical tibial axis (line from knee centre to talar dome centre). Values > 180° indicate valgus alignment; values < 180° indicate varus alignment. interligne angle (IA) shown by the software is the joint‐line angle.

On full‐length standing radiographs (neutral rotation, knees extended), the hip–knee–ankle angle (HKA) was measured as the mechanical femorotibial angle: the angle between the mechanical femoral axis (line from the centre of the femoral head to the knee centre, defined as the midpoint between the tibial spines) and the mechanical tibial axis (line from the knee centre to the centre of the talar dome). This represents direct measurement of the true mechanical axis alignment (Figure 4) [17].

Two fellowship‐trained orthopaedic surgeons performed all measurements independently, each on two occasions; the mean of the two readings was used for primary analyses. Intra and interrater reliability for PTS and HKA were quantified with two‐way random‐effects, absolute‐agreement, single‐measure intraclass correlation coefficients (ICC [2, 1]) with 95% confidence intervals (CIs). Agreement was good to excellent: PTS intra‐rater ICC 0.80 (0.69–0.92) and inter‐rater 0.76 (0.64–0.89); HKA intra‐rater 0.85 (0.82–0.93) and inter‐rater 0.81 (0.73–0.87).

### Outcomes and definitions

Two time‐to‐event endpoints were analysed. ‘Revision‐free survival’ was defined as time from the index procedure to ipsilateral revision ACL reconstruction, with patients not revised censored at their last follow‐up. ‘Global survival’ (composite clinical‐failure–free survival) was defined as time to the earliest of either revision ACL reconstruction or objective graft as prespecified as an instrumented anterior laxity side‐to‐side difference >5 mm on Rolimeter at any postoperative assessment. For cases in which revision followed a prior laxity event, the failure time was taken as the date of the first qualifying laxity assessment (i.e., the earliest event). Patients without either event were censored at the last follow‐up.

Instrumented anterior laxity was measured with a Rolimeter arthrometer (Rolimeter, Jakob Orthopedics) by trained clinicians with the knee flexed ~30° under standardised conditions; side‐to‐side difference (injured minus contralateral) was recorded at 2 months, 1 year and 2 years [1, 5, 7]. Radiographic change was summarised as ΔPTS (postoperative minus preoperative PTS) and ΔHKA (valgus positive). Radiographic union was defined as bridging callus across at least 3 of 4 cortices on anteroposterior and lateral views [16].

Patient‐reported outcomes included: The knee injury and osteoarthritis outcome score (KOOS) (pain, symptoms, activities of daily living, sport/rec and quality of life) [15], The International Knee Documentation Committee (IKDC) subjective knee form was used to assess symptoms, function, and sports activity [9]. The ACL return to sport after injury (ACL‐RSI) scale was used to assess psychological readiness to return to sport [9]. All patient‐reported outcome measures (PROMs) were collected preoperatively and at 2‐year follow‐up.

Prespecified complications were adjudicated at each visit (deep infection/sepsis; residual frontal‐plane instability; iatrogenic varus/valgus or recurvatum; tubercle‐related events; meniscal re‐intervention and nonunion/delayed union). Reoperations were defined as all unplanned returns to the operating room (e.g., revision ACL reconstruction or meniscal procedures); elective hardware removal for irritation/mechanical symptoms was recorded separately and not counted as failure unless accompanied by revision or instability.

### Statistical analysis

Continuous variables were reported as means with standard deviation (SD), and categorical variables as frequencies with percentages. Between‐group comparisons used independent‐samples *t*‐tests (continuous variables) and chi‐square or Fisher's exact tests (categorical variables, when cell counts < 5). Within‐group PROM changes used paired *t*‐tests. Survivorship was analysed using Kaplan–Meier graphs with the log‐rank test.

No formal a priori sample size calculation was performed given the retrospective design and consecutive enrolment. Post‐hoc power analysis indicated that, with the achieved sample sizes, the study had approximately 80% power to detect between‐group Rolimeter differences of ≥1.14 mm at 2 months, ≥1.37 mm at 1 year (*α* = 0.05; pooled SD at 1 year ≈ 2.49 mm), and ≥2.03 mm at 2 years. Missing data (<5% for all outcomes) were handled using complete case analysis (listwise deletion), with no systematic differences in missingness between groups. Significance threshold: *p *< 0.05. All statistical analyses were conducted using JMP Pro (SAS Institute).

## RESULTS

### Survivorship and failure rates

At final follow‐up (mean 27.9 ± 4.4 months), crude revision rates were 5.26% (3/57) for IKO versus 20.0% (10/50) for RKO (*p* = 0.048). When including objective laxity failure (>5 mm side‐to‐side difference), global failure rates were 5.26% (3/57) for IKO versus 20.0% (10/50) for RKO (*p* = 0.048).

**Figure 5:**
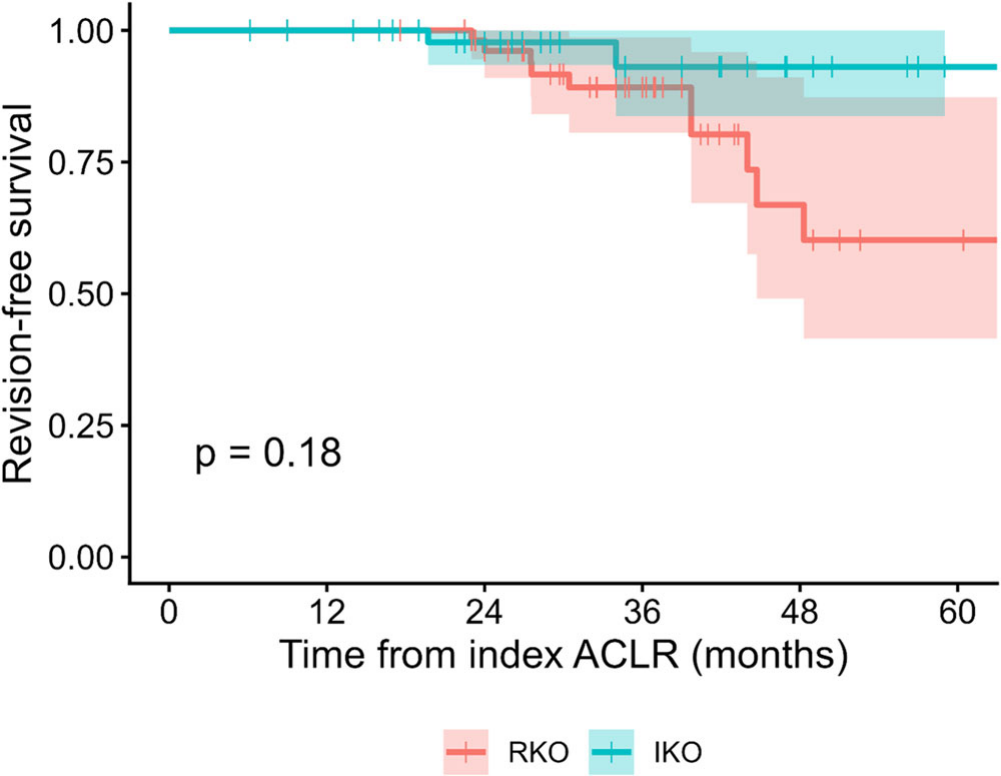
Revision‐free survival. Kaplan–Meier curves for time to ipsilateral revision ACL reconstruction comparing RKO (*n *= 50) and IKO (*n *= 57). One‐, two‐ and five‐year revision‐free survival was 100.0%, 97.7% and 93.1% for IKO versus 100.0%, 96.1% and 60.2% for RKO; median survival was not reached. Between‐group difference by log‐rank test: *p* = 0.180. ACL, anterior cruciate ligament; ACLR, anterior cruciate ligament reconstruction; IKO, infra‐tubercle osteotomy; RKO, retro‐tubercle osteotomy.

**Figure 6:**
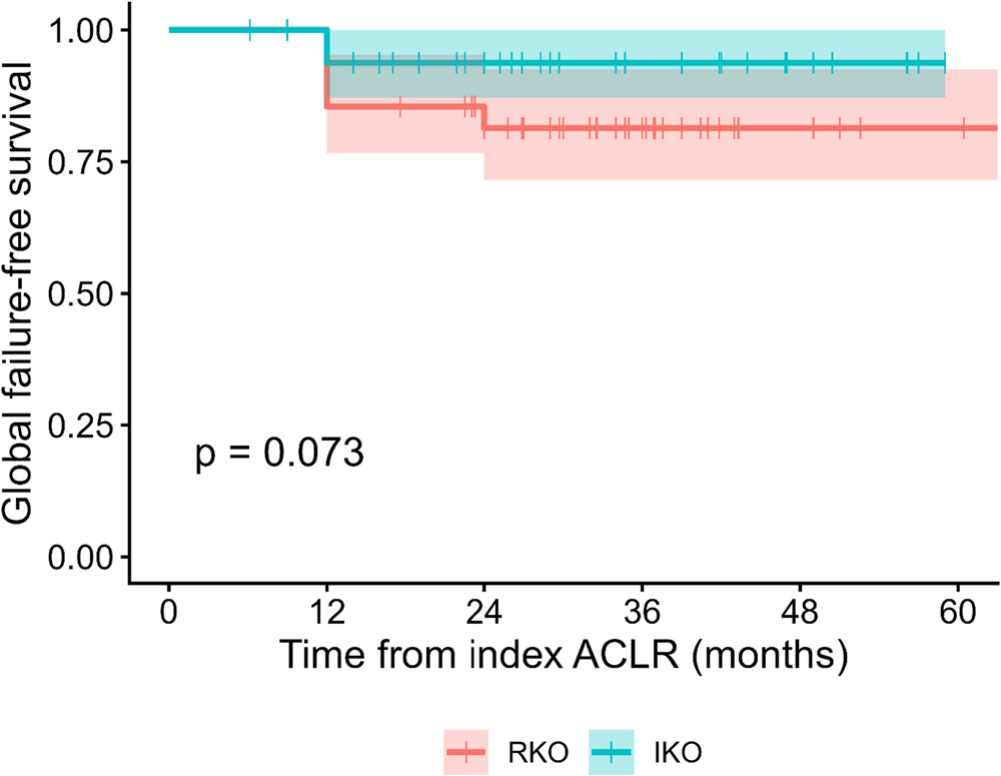
Global survival (composite clinical failure). Kaplan–Meier curves comparing retro‐tubercle RKO (*n* = 50) and infra‐tubercle IKO (*n *= 57). Global survival was defined as time to the earliest of revision ACL reconstruction or objective graft insufficiency producing symptomatic instability (instrumented anterior laxity side‐to‐side difference >5 mm). One‐, two‐ and five‐year survival was 93.8%, 93.8% and 93.8% for IKO versus 85.5%, 81.4% and 81.4% for RKO; median survival was not reached. Between‐group difference by log‐rank test: *p* = 0.073. ACL, anterior cruciate ligament; ACLR, anterior cruciate ligament reconstruction; IKO, infra‐tubercle osteotomy; RKO, retro‐tubercle osteotomy.

Kaplan–Meier survival analysis showed no statistically significant differences between groups for all endpoints (Table 2, Figures 5 and 6). For revision‐only survivorship, 5‐year estimates were 93.1% (IKO) versus 60.2% (RKO) (*p* = 0.180). For the global composite endpoint, 5‐year survivorship estimates were 93.8% for IKO versus 81.4% for RKO (*p *= 0.073). Median survival was not reached in either group.

**Table 2:** Kaplan–Meier survivorship at 1, 2 and 5 years by osteotomy group for two endpoints: Revision‐only and global failure (revision surgery or instrumented laxity >5 mm).

Outcome	Group	Number at risk	1‐year survival (%)	2‐year survival (%)	5‐year survival (%)	Failures (*n*)	Log‐rank *p*
Revision‐only	RKO	50	100.0	96.1	60.2	10	0.180
	IKO	57	100.0	97.7	93.1	3	
Global failure	RKO	50	85.5	81.4	81.4	10	0.073
	IKO	57	93.8	93.8	93.8	3	

*Note*: Values are *S* (%). ‘Number at risk’ is the cohort at time 0 for each endpoint; ‘Failures (*n*)’ counts first events. *P‐*values compare IKO versus RKO by log‐rank test.

Abbreviations: IKO, infra‐tubercle osteotomy; RKO, retro‐tubercle osteotomy.

## PROMS

Both groups demonstrated significant improvements across all KOOS subscales, IKDC and ACL‐RSI scores from baseline to 2‐year follow‐up (all *p* < 0.001; Table 3). When comparing mean improvements (delta scores) between groups, no statistically significant differences were found across any PROM measures (Table 4).

**Table 3:** KOOS outcomes preoperatively and at 2 years by osteotomy group.

PROM	Infra‐tubercle osteotomy			Retro‐tubercle osteotomy		
	Pre‐op mean ± SD	2‐year Mean ± SD	*p* value	Pre‐op Mean ± SD	2‐year Mean ± SD	*p* value
KOOS global	60.4 ± 17.6	85.5 ± 11.7	<0.001	60.2 ± 18.6	84.7 ± 12.0	<0.001
KOOS symptoms	65.0 ± 19.9	85.6 ± 13.4	<0.001	62.8 ± 19.3	83.1 ± 13.8	<0.001
KOOS pain	62.5 ± 20.7	87.5 ± 11.4	<0.001	62.0 ± 19.3	87.8 ± 13.0	<0.001
KOOS ADL	70.2 ± 22.0	90.6 ± 10.0	<0.001	68.9 ± 22.3	90.9 ± 12.8	<0.001
KOOS sport	35.0 ± 20.7	74.2 ± 19.0	<0.001	40.1 ± 21.9	71.8 ± 21.2	<0.001
KOOS QOL	38.3 ± 20.8	76.4 ± 19.3	<0.001	40.0 ± 23.3	70.3 ± 21.6	<0.001

*Note*: Values are mean ± SD. *P‐*values reflect within‐group change from preoperative to 2‐year follow‐up. KOOS is scored 0–100, with higher scores indicating better status.

Abbreviations: ADL, activities of daily living; KOOS, knee injury and osteoarthritis outcome score; PROM, patient‐reported outcome measure; QOL, quality of life; SD, standard deviation.

**Table 4:** Between‐group comparison of 2‐year change (Δ) in KOOS scores.

PROM	Δ IKO (mean ± SD)	Δ RKO (mean ± SD)	*p* value
KOOS global	+24.8 ± 19.4	+24.0 ± 21.3	0.861
KOOS symptoms	+20.1 ± 21.1	+19.5 ± 19.9	0.893
KOOS pain	+24.7 ± 22.5	+25.4 ± 20.2	0.868
KOOS ADL	+20.3 ± 22.9	+21.6 ± 25.1	0.779
KOOS sport	+38.6 ± 26.5	+31.5 ± 28.8	0.211
KOOS QOL	+38.0 ± 27.4	+29.1 ± 29.2	0.122

*Note*: Values are mean ± SD. Positive Δ indicates improvement from baseline; Δ = (2‐year—preoperative). *P‐*values compare IKO versus RKO. KOOS range 0–100 (higher = better). Abbreviations: ADL, activities of daily living; IKO, infra‐tubercle osteotomy; KOOS, knee injury and osteoarthritis outcome score; PROM, patient‐reported outcome measure; QOL, quality of life; RKO, retro‐tubercle osteotomy; SD, standard deviation.

### Radiographic outcomes

Preoperative HKA alignment and PTS were comparable between groups (*p *= 0.60 and *p* = 0.059, respectively). Both techniques achieved substantial PTS correction without significant differences in ΔPTS between groups (−8.2° ± 1.6 for IKO vs. −7.9° ± 1.7 for RKO; *p* = 0.35). However, the RKO group exhibited significantly greater iatrogenic frontal‐plane changes, with a varus shift compared to the IKO group (ΔHKA: −1.32° ± 1.20 vs. +0.45° ± 0.58; *p *< 0.001). Complete radiographic data are presented in Table 5.

**Table 5:** Radiographic alignment (HKA) and posterior tibial slope (PTS) preoperatively and postoperatively, with change from baseline by osteotomy group.

Variable	IKO mean ± SD	RKO mean ± SD	*p* value
HKA preoperative (°)	177.78 ± 2.11	177.57 ± 1.92	0.60
HKA postoperative (°)	177.82 ± 2.08	176.77 ± 1.70	0.005
ΔHKA (°)	+0.45 ± 0.58	–1.32 ± 1.20	<0.001
PTS preoperative (°)	13.73 ± 1.08	13.29 ± 1.28	0.059
PTS postoperative (°)	5.53 ± 1.06	5.40 ± 1.12	0.532
ΔPTS (°)	–8.20 ± 1.61	–7.90 ± 1.74	0.347

*Note*: Values are mean ± SD (degrees). Δ is defined as postoperative−preoperative; positive ΔHKA denotes valgus correction, and negative ΔPTS denotes slope reduction.

Abbreviations: HKA, hip–knee–ankle angle; IKO, infra‐tubercle osteotomy; PTS, posterior tibial slope; RKO, retro‐tubercle osteotomy; SD, standard deviation.

### Anterior laxity

Side‐to‐side laxity measurements were significantly lower in the IKO group compared to the RKO group across all follow‐up timepoints (Table 6). The between‐group difference ranged from 0.9 mm at 2 months (*p* = 0.034) to 1.8 mm at 2 years (*p *= 0.013), with IKO consistently demonstrating lower residual laxity.

**Table 6:** Instrumented anterior tibial laxity (Rolimeter) at 2 months, 1 year and 2 years by the osteotomy group.

Outcome	IKO mean ± SD	RKO mean ± SD	*p* value
Rolimeter at 2 months (mm)	2.4 ± 2.1	3.3 ± 2.0	0.034
Rolimeter at 1 year (mm)	3.5 ± 2.3	4.8 ± 2.7	0.010
Rolimeter at 2 years (mm)	4.0 ± 2.9	5.8 ± 4.2	0.013

*Note*: Values are mean ± SD (mm). *P*‐values compare IKO versus RKO at each time point. Lower values indicate less laxity.

Abbreviations: IKO, infra‐tubercle osteotomy; RKO, retro‐tubercle osteotomy; SD, standard deviatioin.

### Complications

Overall complication rates were low and comparable between groups (14.3% IKO vs. 12.1% RKO, *p *= 0.735). Hardware removal was significantly more frequent in the IKO group (38.8% vs. 17.2%, *p* = 0.016). Time to radiographic bone healing was longer in the IKO group (4.0 ± 0.9 months) compared to the RKO group (3.2 ± 0.6 months, *p *< 0.0001). Complete complication data are presented in Table 7.

**Table 7:** Complications, hardware removal and time to bone healing by the osteotomy group.

Outcome	IKO (*n* = 50)	RKO (*n *= 57)	*p* value
Overall complication rate (%)	14.3%	12.1%	0.735
Hardware removal rate (%)	38.8%	17.2%	0.016*
Time to bone healing (months)	4.0 ± 0.9	3.2 ± 0.6	<0.001*
Meniscus injuries	6.12%	1.72%	0.226

*Note*: Values are % or mean ± SD (months). *P*‐values compare IKO versus RKO.

Abbreviations: IKO, infra‐tubercle osteotomy; RKO, retro‐tubercle osteotomy; SD, standard deviation.

* *p* < 0.05.

## DISCUSSION

The most important finding of the present study was that IKO achieved comparable survivorship to retro‐tubercle technique while demonstrating better preservation of coronal alignment and significantly lower residual anterior laxity at all postoperative timepoints.

The most clinically significant finding is the low overall crude failure rate in both groups compared to historical revision ACLR outcomes without slope correction. The crude revision rates of 6.0% (IKO) and 20.0% (RKO) contrast with reported failure rates for second and third revision ACLR in cohorts without slope correction. Trojani et al. documented 37% failure at 4.5‐year follow‐up in second revisions [19], while Wright et al.'s systematic review reported 8%–29% failure rates, with higher rates in multiple‐revision scenarios [21]. Given that this cohort consisted predominantly of second (73.5%–82.8%) and third (17.2%–24.5%) revision cases, the combined crude failure rate of 13% falls below these benchmarks. However, direct comparisons are limited by differences in follow‐up duration, case mix and patient populations. These findings suggest that addressing elevated PTS may alter the failure trajectory in high‐risk revision scenarios, though randomised comparisons are needed to confirm this hypothesis.

Both infra‐tubercle and retro‐tubercle techniques effectively restored knee stability, as demonstrated by normalisation of Rolimeter side‐to‐side anterior translation at 1 and 2 years postoperatively. However, IKO was associated with significantly lower side‐to‐side anterior knee laxity at all postoperative timepoints compared to the RKO (differences of 0.9 mm at 2 months, 1.3 mm at 1 year, and 1.8 mm at 2 years; all *p *< 0.05). While these differences are statistically significant, the clinical significance of a 1–2 mm difference in instrumented laxity remains uncertain and warrants further investigation. One possible explanation is that by positioning the wedge/hinge distal to the tubercle, the infra‐tubercle approach may focus correction more purely in the sagittal plane while preserving patellar height and the quadriceps lever arm. Guo and Smith et al. recently demonstrated that alterations in PTS influence joint mechanics including patellar tracking and extensor mechanism loading [6]. The infra‐tubercle technique, by maintaining the native tubercle position and minimising disruption to the extensor mechanism insertion, may better preserve these biomechanical relationships and contribute to improved anterior stability.

Both methods achieved a substantial reduction in PTS (ΔPTS −8.20 ± 1.61° for IKO vs. −7.90 ± 1.74° for RKO, *p *= 0.35). However, a notable finding was that the infra‐tubercle technique better preserved frontal plane alignment than the retro‐tubercle technique, as measured by the HKA angle (ΔHKA +0.45 ± 0.58° for IKO vs. −1.32 ± 1.20° for RKO, *p* < 0.001). This finding is consistent with Patel et al., who reported a mean increase of 1.4° in the retro‐tubercle group in a similar cohort (mean age: 20.4 years, mean follow‐up: 26.7 months) [14]. The clinical implications of this iatrogenic varus shift remain unclear but may be relevant in patients with pre‐existing varus alignment or medial compartment disease. The infra‐tubercle approach, by positioning the wedge and hinge distal to the tubercle, may focus corrective forces more purely in the sagittal plane, minimising unintended coronal plane effects that could arise from the ascending posterior cuts used in the retro‐tubercle technique. Though, statistically significant it remains unclear how clinically significant <2.0° varus alignment is, as previous studies have demonstrated that >5° of coronal malalignment may effect outcomes following ACLR [18].

Both techniques had acceptable safety profiles, though each has distinct considerations. No cases of nonunion occurred in either group, and all osteotomies healed, but with a statistically significant difference in healing time (4.0 ± 0.9 vs. 3.2 ± 0.6 months, *p* < 0.0001); however, this difference of less than one month is unlikely to be clinically significant. A notable finding was the need for elective hardware removal: 38.8% of IKO patients versus 17.2% of RKO patients (*p* = 0.016) underwent plate or screw removal due to local irritation. This is consistent with other slope‐HTO studies reporting high rates of symptomatic hardware. Vivacqua et al. documented 26% reoperations for hardware removal after closing‐wedge slope osteotomies [20]. Several factors may explain the higher rate in IKO: (1) the distal position of the plate may increase prominence and soft tissue irritation with kneeling or direct contact; (2) the young, active patient population may be more symptomatic from hardware presence; (3) technique‐related factors such as plate contouring or screw trajectory may differ between approaches. Given the young, active population often undergoing these procedures, patients should be counselled that a secondary hardware removal procedure may be anticipated, particularly with the infra‐tubercle technique.

### Clinical relevance

These findings have direct clinical implications for revision ACL reconstruction planning. Surgeons may consider IKO when coronal alignment preservation is critical or in patients with baseline varus alignment or medial compartment concerns. The lower residual laxity with IKO, although modest in absolute magnitude (1–2 mm), may be relevant in high‐demand athletes or multiple‐revision scenarios where every millimetre of stability matters. Conversely, the shorter healing time and lower hardware removal rate with RKO may be advantageous when expedited return to activity is prioritised or when patient preference strongly favours avoiding secondary procedures.

Regardless of technique selection, patients should be counselled that slope‐reducing osteotomy substantially improves revision ACLR outcomes compared to historical controls without slope correction and that hardware removal may be necessary, particularly with the infra‐tubercle technique (approximately 40% vs. 17%). Shared decision‐making incorporating patient activity demands, alignment status, prior surgical history, and tolerance for secondary procedures should guide technique selection. Both techniques represent valuable tools in the armamentarium for managing complex revision ACL scenarios with elevated PTS.

### Limitations

This study has several limitations. The sample size may be insufficient to detect small differences between IKO and RKO groups, as demonstrated by being underpowered for survival analysis despite detecting differences in crude failure rates. The comparisons were retrospective in nature, with the osteotomy technique determined by surgeon preference and temporal evolution of practice rather than randomisation, introducing potential selection bias. The temporal shift from predominantly RKO (early study period, 2016–2019) to IKO (later period, 2020–2024) may have introduced confounding related to surgical experience, patient selection, or other unmeasured factors. This temporal pattern potentially favours the IKO group due to surgeon learning curve effects and refinement of technique over time. Additionally, the RKO group had a significantly lower rate of prior meniscectomy (27.6% vs. 51.0%, *p* = 0.01), a potential confounder as meniscal deficiency may influence revision risk and knee stability [13]. This baseline imbalance could bias results against the IKO group, potentially underestimating the true benefit of the infra‐tubercle technique. The single‐surgeon design strengthens internal validity but limits external generalisability. Whether these findings translate to other surgeons and practice settings requires validation. Second, the follow‐up duration (mean 27.9 ± 4.4 months) may be insufficient to capture late failures or long‐term degenerative changes. Longer‐term follow‐up would be beneficial to determine if the initial graft protection and functional improvements are maintained and to monitor for osteoarthritic changes, particularly relevant in this young study population. Finally, as with all multicomponent surgeries, it is challenging to isolate the effect of the osteotomy from the revision ACLR itself or concomitant procedures such as LET. Despite these limitations, this study is one of the first comparative analyses of infra‐versus retro‐tubercle slope HTO in ACL revision, and the results provide valuable evidence in a relatively new area. Future studies should include randomised controlled trials with longer follow‐up, multicentre validation, and cost‐effectiveness analyses comparing the two techniques.

## CONCLUSION

Infra‐tubercle and retro‐tubercle slope‐reducing HTO are both effective adjuncts to revision ACL reconstruction in patients with excessive PTS. The present findings demonstrate that IKO achieves outcomes at least equivalent to the retro‐tubercle technique, with potential advantages including better preservation of coronal alignment and lower residual anterior laxity. These benefits come at the trade‐off of more frequent elective hardware removal and slightly longer time to union.

## AUTHOR CONTRIBUTIONS

All authors contributed equally to the conception, design, data collection, analysis, drafting and critical revision of this manuscript. All authors approved the final submitted version.

## CONFLICT OF INTEREST STATEMENT

Matthieu Ollivier is a paid consultant and receives royalties from Newclip. Matthieu Ollivier is also a paid consultant and receives royalties from Stryker. The remaining authors declare no conflicts of interest.

## ETHICS STATEMENT

Please include the name of the Institutional Review Board (IRB) and the approval number. If not applicable, please state so. Ethics approval was obtained from the Institute of Movement and Musculoskeletal System, Department of Orthopaedic Surgery of Sainte‐Marguerite Hospital, France. This study was approved by the institutional review board (IRB#Cse_pads25_043_dgr). Informed consent was obtained from all patients involved in this study.

## Data Availability

All data used in this study are available from the corresponding author upon reasonable request.
